# Hospital admissions due to alcohol related disorders among young adult refugees who arrived in Sweden as teenagers – a national cohort study

**DOI:** 10.1186/s12889-017-4645-5

**Published:** 2017-08-08

**Authors:** Hélio Manhica, Karl Gauffin, Ylva B. Almquist, Mikael Rostila, Lisa Berg, Ainhoa Rodríguez García de Cortázar, Anders Hjern

**Affiliations:** 1 Centre for Health Equity Studies (CHESS), Karolinska Institutet/Stockholm University, Sveavägen 160, SE-106 91 Stockholm, Sweden; 2 Andalusian School of Public Health (EASP), 18011 Granada, Spain; 3 Department of Medicine, Clinical Epidemiology, Karolinska Institutet, 171 77 Stockholm, Sweden

**Keywords:** Young adult refugees, Hospital care, Alcohol related disorders, Migration, Culture

## Abstract

**Background:**

Psychological distress and lack of family support may explain the mental health problems that are consistently found in young unaccompanied refugees in Western countries. Given the strong relationship between poor mental health and alcohol misuse, this study investigated hospital admissions due to alcohol related disorders among accompanied and unaccompanied young refugees who settled in Sweden as teenagers.

**Methods:**

The dataset used in this study was derived from a combination of different registers. Cox regression models were used to estimate the risks of hospital care due to alcohol related disorders in 15,834 accompanied and 4376 unaccompanied young refugees (2005–2012), aged 13 to 19 years old when settling in Sweden and 19 to 32 years old in December 2004. These young refugees were divided into regions with largely similar attitudes toward alcohol: the former Yugoslavian republics, Somalia, and the Middle East. The findings were compared with one million peers in the native Swedish population.

**Results:**

Compared to native Swedes, hospital admissions due to alcohol related disorders were less common in young refugees, with a hazard ratio (HR) of 0.65 and 95% confidence interval (CI) between 0.56 and 0.77. These risks were particularly lower among young female refugees. However, there were some differences across the refugee population. For example, the risks were higher in unaccompanied (male) refugees than accompanied ones (HR = 1.49, 95% CI = 1.00–2.19), also when adjusted for age, domicile and income. While the risks were lower in young refugees from Former Yugoslavia and the Middle East relative to native Swedes, independent of their length of residence in Sweden, refugees from Somalia who had lived in Sweden for more than ten years showed increased risks (HR = 2.54, 95% CI = 1.71–3.76), after adjustments of age and domicile. These risks decreased considerably when income was adjusted for.

**Conclusion:**

Young refugees have lower risks of alcohol disorders compared with native Swedes. The risks were higher in unaccompanied young (male) refugees compared to the accompanied ones. Moreover, Somalian refugees who had lived in Sweden for more than ten years seems to be particularly vulnerable to alcohol related disorders.

## Background

Epidemiological studies have consistently reported poor mental health among refugees in Western countries [1, 2]. This has been particularly applicable to unaccompanied young refugees, who face higher risks of psychological distress and lack the social and emotional support of their friends and family members [3, 4]. This vulnerability has been particularly associated with health-risk behaviours such as substance misuse as coping strategies to deal with stressors [5, 6].

Previous studies have shown that alcohol related disorders vary significantly in the heterogeneous migrant population in Sweden [7, 8]. Thus, the importance of norms or attitudes to alcohol intake in the refugees’ and migrants’ country of origin has attracted considerable interest, particularly as alcohol is culturally prohibited in Muslims, especially women [2, 9, 10]. However, their lower consumption rates rather may also be a reflection of lack of opportunities to buy alcohol.

Despite this, heavy alcohol consumption has been reported among marginalised East African refugees in Australia [11], Israel [12], and males with Arabic backgrounds facing unemployment and criminal involvements in the US [13]. These studies suggest that socioeconomic adversity and exposure to psychological distress [14] might play an important role in the aetiology of alcohol misuse. Thus, through self-medication, migrants and refugees are believed to misuse alcohol and illicit substances as a way to relieve painful feelings resulting from war related traumas, socioeconomic exclusion, and different forms of acculturative stress [15, 16].

Alcohol misuse is a worldwide problem as it brings negative consequences for the individuals and society as a whole, causing millions of deaths and injuries. It has been associated with deterioration of mental health, incite violence, suicide attempts, social exclusion, criminality and deterioration of health status [17]. In Sweden, alcohol related disorders are responsible for about 2000 deaths annually [18]. Outpatient and inpatient treatment for alcohol disorders in Sweden includes a combination of psychosocial interventions (twelve-step facilitation therapy, cognitive behavioural therapy and motivating conversation) and drug treatments (nalmefene, naltrexone, akamprosat and acamprosate). The treatment goal for alcohol related disorders in Sweden is individual and depends of the motivation of the patient [19].

Increasing numbers of young refugees have arrived in Sweden with one or both of their parents since the middle of the 1970s [2]. The numbers of unaccompanied minors has also increased rapidly over the last decade, reaching 33,000 in 2015, and they have primarily been 15 to 17-year-old boys from war-torn countries like Somalia, Afghanistan, Iran, Syria and Iraq [20]. Apart from the psychological distress they have experienced in their country of origin [1], young refugees are often exposed to severe socio-environmental stressors in Sweden, such as discrimination, long-term unemployment, low income, and living in substandard housing in deprived socioeconomic neighbourhoods [2, 21, 22]. These pose additional risk factors for poor mental health and health behaviours, including alcohol misuse. Unaccompanied young refugees might be more vulnerable than accompanied ones as they might face dual experiences of psychological and acculturative stress distress, and lack of social and emotional support from their families.

The current study takes its point-of-departure in the risk of hospitalisation due to alcohol related disorders among young male and female refugees who arrived to Sweden as teenagers, as compared to the majority population. Since young refugees constitute an exceedingly heterogeneous group, we also explore whether the risks of alcohol related disorders are different between accompanied and unaccompanied refugees and whether the risks vary according to region of birth and length of residence.

## Methods

Sweden has a long tradition of national registers, with health and socioeconomic indicators that can be linked to each other through the unique 10-digit personal identification number (PIN) that follows every Swedish resident from birth or immigration to death. The datasets are anonymous and the researchers have no access to any personal information that could identify individuals included in the datasets. The Swedish national registers are protected by special legislation, which makes it possible for researchers to collect certain information without personal consent [23]. The Regional Ethics Committee in Stockholm approved the study before any records were linked (decision number 2013/811–31/5).

### Study groups

The study population consisted of individuals born between 1972 and 1985, who were alive and resident in Sweden according to the Register of the Total Population on 31 December 2004. The refugee population comprised all young individuals who were between 13 and 19 years of age when they received permanent residency in Sweden as refugees or because they were related to a refugee. It also included individuals who were granted residence permits in Sweden on humanitarian grounds. Young refugees who had obtained residency in Sweden because they were related to a family member who was also a refugee were categorized as accompanied. Young refugees who arrived alone, without the company of at least one parent or an adult who by law or custom was responsible for their care, were categorized as unaccompanied young refugees.

The refugee population was acquired from STATIV, a longitudinal database for integration studies, which is held by Statistics Sweden and based on data from the Board of Migration. This population was chosen to include individuals coming from countries and regions that included significant numbers of unaccompanied and accompanied young refugees that shared similar principles and attitudes towards patterns and levels of alcohol consumption, according to the World Health Organization [24]. The largest number of refugees came from the former Yugoslavian republics (*n* = 9776) and the majority of these were Muslims from Bosnia Herzegovina [25]. The other refugees were from Somalia (*n* = 2372) and the Middle East (*n* = 8062) including Iran and Iraq. The native population consisted of 1,009,027 individuals with both parents born in Sweden.

### Outcome

The outcome variable was defined as at least one register entry on alcohol related medical care, with a diagnosis of an alcohol related psychiatric or medical disorder, as well as alcohol related mortality from 1 January 2005 to 31 December 2012. Data on alcohol related disorders were collected from the National Patient Register [26] and data on alcohol related mortality were collected from the National Cause of Death registers. Medical care for alcohol related hospitalizations that did not involve long-term alcohol misuse, such as unspecified alcohol use with intoxication (ICD 92), were excluded.

### Covariates

The socio-demographic covariates of age, gender, disposable income, and domicile were retrieved in January 2005, from the Longitudinal Integration Database for Health Insurance and Labour Market Studies. *Domicile* was split into three categories and defined by the place of residence in 2008: a big city referred to the metropolitan areas of Sweden’s three largest cities, Stockholm, Gothenburg and Malmo, a town covered other predominately urban communities and rural covered the remainder. *Disposable income* was divided into quintiles, which included all registered sources of income after taxes and was then divided by the consumer units in the household, according to a formula developed by Statistics Sweden.

### Statistical analysis

We used Cox-regression models to estimate hazard ratios (HRs) of hospital admission due to alcohol related disorders. As a first step (Table 2), we estimated HRs of hospital admissions for young refugees compared with native Swedes. Models 1a and 1b adjusted for age, domicile and gender, whereas income was added in Models 1b and 2b. Furthermore, we carried out interaction analyses of gender and native Swedes/young refugees on the risks of hospital admission. A statistically significant interaction effect of gender and country and region of origin on the outcome was found. We therefore decided to stratify the remaining analyses by gender. As a second step, Table 3 compares the HRs in unaccompanied and accompanied young with native Swedes as reference group in male and female separately, after adjustments of age and domicile in Models 1a and 2a. Income was added in Models 1b and 2b. We also show the results with the accompanied young refugees as the reference group and used the same adjustment procedure. As a third step, we compared the risks of hospital admission across the regions of origins of the refugee population with native Swedes, separately for male and female (Table 4) Models 1a and 2a were adjusted for age and domicile and Models 1b and 2b were further adjusted for income. Furthermore, we performed additional analysis based on how long the young refugees had lived in Sweden, differentiating between groups who had lived in Sweden for more or less than 10 years. We used the adjustments procedure outlined above.

## Results

The socioeconomic characteristics of the study population are presented in Table 1. The majority of the teenage refugees were male and the unaccompanied refugees from the Middle East and Somalia were slightly older than the accompanied refugees. In general, the accompanied refugees had not had a residence permit in Sweden as long as the unaccompanied refugees had. The refugees were more likely to live in big cities, such as the metropolitan areas of Sweden’s three largest cities: Stockholm, Gothenburg and Malmo. When the distribution of disposable income was divided into quintiles, it showed that refugees from Somalia and the Middle East were overrepresented among those with the lowest disposable income.

**Table 1:** Socio-demographic indicators of the study population (*n* = 1,029,233)

Variable	Sweden	F. Yugoslavia		Somalia		Middle East	
		*Acc.*	*Unacc.*	*Acc.*	*Unacc.*	*Acc.*	*Unacc.*
	*1,009,027*	*8301*	*1475*	*1088*	*1284*	*6445*	*1617*
Age	*Mean (SD)*	*Mean (SD)*	*Mean (SD)*	*Mean (SD)*	*Mean (SD)*	*Mean (SD)*	*Mean (SD)*
Mean age (SD)	25.6	24.9	26.9	24.1	27.1	23.7	26,1
	*%*	*%*	*%*	*%*	*%*	*%*	*%*
Gender							
Male	51.5	52.1	53.7	56.4	61.3	54.1	73.3
Residency (years)							
0–10	-	71.7	43.8	65.2	52.3	68.3	54.3
> 10 years	-	28.3	56.2	34.8	47.4	31.7	45.7
Domicile							
Big city	43.9	49.3	51.3	69.4	75.1	58.4	61.9
Town	42.9	45.4	42.4	27.9	23.2	38.7	35.5
Rural	13.2	5.3	6.3	2.7	1.7	2.9	2.6
Income (Quintiles)							
1 (lowest)	11.2	26.1	23.7	58.7	45.9	54.9	37.9
2	17.2	24.4	25.4	19.1	24.3	18.7	21.4
3	21.3	22.1	21.7	9.2	12.0	11.4	14.4
4	24.5	18.2	16.5	8.9	11.6	8.3	13.1
5 (highest)	25.8	9.2	12.7	4.1	6.2	6.6	13.1

*SD* Standard Deviation, *Acc* Accompanied, *Unacc* Unaccompanied; Sweden refers to native Swedish population

Table 2 shows the crude rates and differences in the HRs of hospital care due to alcohol related disorders. The rates of hospital admission among native Swedes were 1.1%, as opposed to 0.8% in young refugees. Table 2 also shows that the risks of hospital admission were lower among young refugees (HR = 0.65, 95% CI = 0.56–0.77) compared with native Swedes after adjustments of age, gender, and domicile in Model 1a. The risks further decreased (HR = 0.48, 95% CI = 0.40–0.57) when income was further adjusted for in Model 1b. The interaction analysis indicated even lower risks of hospital admission in female young refugees compared with male refugees after adjustments of age and domicile in the Model 2a (HR = 0.58, 95% CI = 0.39–0.87) and income in Model 2b (HR = 0.51, 95% CI = 0.41–0.93).

**Table 2:** Rates and hazard ratios of alcohol related disorders among young refugees compared to native Swedes (n: 1,029,233)

Country/region of origin			Cox regression models			
	N	Cases: N (%)	HR (95% CI)	HR (95% CI)	HR (95% CI)	HR (95% CI)
			Model 1a	Model 1b	Model 2a	Model 2b
Native Swedes^a^	1,009,027	11,265 (1.1)	1	1	1	1
Young refugees	20,210	151 (0.8)	0.65 (0.56–0.77)	0.48 (0.40–0.57)	0.75 (0.62–0.90)	0.54 (0.45–0.65)
Young refugee x Female ^b^					0.58 (0.39–0.87)	0.51 (0.41–0.93)

Model 1a & 2a: adjusted for age, domicile, and gender

Model 1b & 2b: adjusted for age, domicile, gender, and income

^a^Reference category

^b^ Interaction term: Young refugee * Female

Table 3 compares rates between native Swedes and accompanied and unaccompanied young refugees, stratified by gender. The rates of hospital admission due to alcohol related disorders reported in Model 1a and Model 1b were significantly lower among accompanied young refugees compared to native Swedes, among males (HR = 0.68, 95% CI = 0.55–0.85; HR = 0.68, 95% CI = 0.51–0.92) and females alike (HR = 0.37, 95% CI = 0.24–0.56; HR = 0.28, 95% CI = 0.18–0.42). However, the hazard ratios for unaccompanied male (HR = 0.98, 95% CI = 0.71–1.35; HR = 0.43, 95% CI) and female (HR = 0.70, 95% CI = 0.34–1.39; HR = 0.50, 95% CI = 0.25–1.01) refugees were overall more similar, and not statistically significantly different, to those of native Swedes. In Model 2a and Model 2b, the reference category has been alternated to test the differences between unaccompanied and accompanied young refugees. The results indicate that unaccompanied young male and female refugees have higher risks of hospitalization due to alcohol related disorders compared to their accompanied counterparts. The fact that these differences are not statistically significant is mainly due to power issues (i.e. a limited number of cases in the group of unaccompanied refugees).

**Table 3:** Rates and hazard ratios for alcohol related disorders among unaccompanied and accompanied young refugees compared to native Swedes in male and female young refugees (n: 1,029,233)

Country/region of origin			Cox regression models			
	N	Cases: N (%)	HR (95% CI)	HR (95% CI)	HR (95% CI)	HR (95% CI)
			Model 1a	Model 1b	Model 2a	Model 2b
Male						
Native Swedes^a^	519,674	7503 (1.4)	1	1	1.45 (1.16–1.80)	2.00 (1.60–2.50)
Unaccompanied	2765	39 (1.4)	0.98 (0.71–1.35)	0.43 (0.35–0.51)	1.43 (0.97–2.10)	1.49 (1.00–2.19)
Accompanied^b^	8418	82 (1.0)	0.68 (0.55–0.85)	0.68 (0.51–0.92)	1	1
Female						
Native Swedes^a^	489,353	3762 (0.8)	1	1	2.67 (1.75–4.07)	3.56 (2.34–5.43)
Unaccompanied	1611	8 (0.5)	0.70 (0.34–1.39)	0.50 (0.25–1.01)	1.86 (0.82–4.17)	1.81 (0.80–4.07)
Accompanied^b^	7416	22 (0.3)	0.37 (0.24–0.56)	0.28 (0.18–0.42)	1	1

Model 1a & 2a: adjusted for age, and domicile

Model 1b & 2b: adjusted for age, domicile, and income

^a^Reference category for Model 1a and Model 1b

^b^Reference category for Model 2a and Model 2b

**Table 4:** Rates and hazard ratios of alcohol related disorders among young refugees and native Swedish, by country/region of origin and length of residence in male and female young refugees (n: 1,029,233)

				Cox regression models			
Country/region of origin				Male		Female	
	N	Male Cases: N (%)	Female Cases: N (%)	HR (95% CI) Model 1a	HR (95% CI) Model 1b	HR (95% CI) Model 2a	HR (95% CI) Model 2b
Native Swedes ^a^	1,009,027	7503 (1.4)	3762 (0.8)	1	1	1	1
F. Yugoslavia	9776	36 (0.7)	9 (0.2)	0.49 (0.35–0.69)	0.40 (0.28–0.56)	0.25 (0.13–0.48)	0.20 (0.10–0.39)
Somalia	2372	41 (2.9)	7 (0.7)	2.17 (1.59–2.98)	1.49 (1.08–2.07)	1.03 (0.49–2.16)	0.71 (0.33–1.49)
Middle East	8062	44 (0.9)	14 (0.4)	0.66 (0.49–0.88)	0.46 (0.34–0.61)	0.50 (0.29–0.85)	0.35 (0.20–0.59)
Length of residence by origin							
Native Swedes ^a^				1	1	1	1
F. Yugoslavia 0–10 years of residence	6600	30 (0.9)	5 (0.2)	0.61 (0.42–0.87)	0.48 (0.33–0.69)	0.20 (0.08–0.48)	0.15 (0.06–0.38)
F. Yugoslavia >10 years of residence	3176	6 (0.4)	4 (0.3)	0.26 (0.11–0.57)	0.22 (0.09–0.49)	0.36 (0.14–0.97)	0.31 (0.11–0.83)
Somalia 0–10 years of residence	1384	20 (2.6)	2 (0.3)	1.90 (1.21–2.98)	1.36 (0.86–2.13)	0.42 (0.10–1.68)	0.29 (0.07–1.17)
Somalia >10 years of residence	988	21 (3.3)	5 (1.4)	2.52 (1.62–3.92)	1.68 (1.05–2.66)	2.43 (1.01–5.87)	1.65 (0.68–3.97)
Middle East 0–10 years of residence	5279	24 (0.8)	7 (0.3)	0.56 (0.37–0.83)	0.37 (0.25–0.56)	0.34 (0.16–0.72)	0.22 (0.10–0.48)
Middle East >10 years of residence	2783	20 (1.2)	7 (0.6)	0.86 (0.55–1.32)	0.62 (0.39–0.98)	0.96 (0.45–2.02)	0.74 (0.35–1.55)

Model 1a & 2a: adjusted for age and domicile

Model 1b & 2b: adjusted for age, domicile, and income

^a^Reference category

The upper part of Table 4 shows the risks of hospital admission due to alcohol related disorders in male and female young refugees by specific region of origin, with native Swedes as the reference category. According to Model 1a, the HRs of hospital care were lower in young male refugees from the former Yugoslavian republics and Middle East when compared with native Swedes males. Male refugees from Somalia showed a different pattern (HR = 2.17, 95% CI = 1.59–2.98), when adjusted for age and domicile in Model 1a. These risks however decreased considerably (HR = 1.49, 95% CI = 1.08–2.07), when income was adjusted for in Model 1b. The risks of hospital care were generally lower in female refugees compared with native Swedish women regardless of adjustments for age, domicile, and income.

In the lower part of Table 4, the associations between region of origin and hospital admission are further divided by length of residence. Overall, the decreased risks among male and female refugees from former Yugoslavia are particularly low among those who resided in Sweden for more than 10 years. The opposite is true for young refugees from the Middle East. Among the refugees from Somalia, both males and females who had been granted residence permits in Sweden more than 10 years before had exceedingly high risks of hospital admission due to alcohol related disorders (HR = 2.52, 95% CI = 1.64–3.92; HR = 2.43, 95% CI), when adjusted for age and domicile (Model 1a). However, these risks reduced considerably when adjusted for income in Model 1b (HR = 1.68, 95% CI = 1.05–2.66). The results also suggested low risks of hospital care due to alcohol disorders in young refugees from the former Yugoslavian republics and Middle East, regardless of length of residence and adjustment procedures.

## Discussion

This register based follow-up study of hospital admission due to alcohol related disorders among young refugees who were aged 13 to 19 years when they settled in Sweden, indicated that young male and female refugees had lower risks of hospital admission due to alcohol related disorders than native Swedes. As expected, the heterogeneity of the refugee population was reflected in the risks for alcohol related disorders. For example, the risk of hospitalisation due to alcohol related disorders among unaccompanied young refugees was higher compared to the accompanied ones, largely on par with the risks for native Swedes. Moreover, while young male and female refugees from Former Yugoslavia and the Middle East had lower risks of hospitalisation due to alcohol related disorders relative to native Swedes, independent of their length of residence in Sweden, Somalian refugees who had lived in Sweden for more than ten years showed increased risks.

The high hospitalization risks found among unaccompanied refugees – as compared to the accompanied ones – might have been associated with higher psychological distress and lack of social and emotional support from their families. This could have left them feeling alone when it came to coping with traumatic experiences that happened in their country of origin and during the migration process [27, 28]. Unaccompanied refugees may also have lacked the social control they needed to avoid risky behaviours, such as the positive role models provided by their parents or another adult family member. This could have meant that they adapted more easily to the culture of drinking in Sweden than children who arrived with their parents. In addition, unaccompanied young refugees might also lack emotional support to deal with cumulative integration challenges caused by cultural clashes, discrimination and socioeconomic exclusion. This hypothesis is in line with the self-medication assumption, namely that due to their high vulnerability, unaccompanied refugees were more likely to misuse alcohol as a coping strategy to deal with their traumatic memories and the challenges of integrating into Swedish society [5, 6].

The results from our study indicated that the risks of hospitalization were, in general, lower among the refugee population than native Swedish peers, with the exception of male and female refugees from Somalia who had resided in Sweden for more than 10 years. The high risk for alcohol related disorders in this particular group from Somalia was contrary to our expectations, as it did not confirm the protective effect of religion and norms on alcohol intake formed in the refugees’ countries of origin [24]. It is possible that their vulnerability to alcohol misuse might have been associated with their higher psychiatric morbidity along with their higher long-term exposure to socioeconomic disadvantages [21, 22, 29].

Hospitalization due to alcohol related disorders implies serious morbidity related to alcohol misuse and it is therefore possible that the increased risks of hospitalization found in young refugees who moved from Somalia to Sweden might reflect the deterioration of their psychological and socioeconomic living conditions over time. Although the risks of hospitalization increased with the number of years they had lived in Sweden after gaining legal residency, these risks diminished when we adjusted for income. This may be in line with previous studies that addressed the importance of living conditions when explaining the high rates of heavy drinking among socioeconomically disadvantaged refugees from East Africa who settled in Australia and Israel [11, 12]. In general, our findings call for further investigations that would also apply qualitative methods to investigate the relationships between acculturation, attitudes to alcohol consumption and socioeconomic living conditions in this vulnerable population.

When it came to gender differences, the lower prevalence among refugees compared to native Swedes was more pronounced among women than among men. These results agreed with previous findings that reported low rates of alcohol misuse among non-European migrants [7, 30] and stressed the importance of conservative attitudes about alcohol intake in female Muslim migrants. However, the lower risks of alcohol related disorders in female refugees compared with their male counterparts should be interpreted with caution as due to the lower number of female refugees compared with male, especially among unaccompanied young refugees.

The major strength of our study was that we were able to study a whole national cohort of immigrants who had either been granted permanent residency as refugees in their own right or because they were related to refugees. They all arrived in Sweden in their teens, which is potentially a very vulnerable period for migration. Furthermore, we were able to create a proxy variable that attempted to distinguish between teenagers who settled in Sweden with their families and those who were unaccompanied.

Despite these strengths, our study had some limitations. First, it was possible that not all young refugees coming from countries dominated by Muslim culture were actually Muslims and our results should be interpreted with caution with regard to this. For example, refugees coming from the former Yugoslavian republics included Muslims, Christians, although Bosnian Muslims made up the greater proportion of this population. In addition, residency was based on the date when legal residency was granted, not the date when the person entered Sweden. As a consequence, it is important to consider this when interpreting the results, since it is possible that some refugees could have lived in Sweden for longer than estimated.

## Conclusion

Young refugees have in general lower risks of hospitalization due to alcohol related disorders compared with native Swedes**.** Unaccompanied young refugees seemed more vulnerable than accompanied ones. These risks appear to differ within the refugee population, since they were more marked among refugees from Somalia who had a longer duration of legal residence in Sweden. The relationship between socioeconomic disadvantages and alcohol related disorders in young refugees merits further investigation.
